# Antimicrobial spray nanocoating of supramolecular Fe(III)-tannic acid metal-organic coordination complex: applications to shoe insoles and fruits

**DOI:** 10.1038/s41598-017-07257-x

**Published:** 2017-08-01

**Authors:** Ji Hun Park, Sohee Choi, Hee Chul Moon, Hyelin Seo, Ji Yup Kim, Seok-Pyo Hong, Bong Soo Lee, Eunhye Kang, Jinho Lee, Dong Hun Ryu, Insung S. Choi

**Affiliations:** 1 Center for Cell-Encapsulation Research, Department of Chemistry, KAIST Daejeon, 34141 Korea; 2 Startup KAIST, KAIST Daejeon, 34141 Korea; 3 HC Lab, 235 Creation Hall, 193 Munji Road, Daejeon, 34051 Korea

## Abstract

Numerous coating strategies are available to control the surface properties and confer new properties to substrates for applications in energy, environment, biosystems, etc., but most have the intrinsic limitations in the practical setting: (1) highly specific interactions between coating materials and target surfaces are required for stable and durable coating; (2) the coating of bulk substrates, such as fruits, is time-consuming or is not achievable in the conventional solution-based coating. In this respect, material-independent and rapid coating strategies are highly demanded. We demonstrate spray-assisted nanocoating of supramolecular metal-organic complexes of tannic acid and ferric ions. The spray coating developed is material-independent and extremely rapid (<5 sec), allowing for coating of commodity goods, such as shoe insoles and fruits, in the controlled fashion. For example, the spray-coated mandarin oranges and strawberries show significantly prolonged post-harvest shelf-life, suggesting practical potential in edible coating of perishable produce.

## Introduction

Nanometer-thick coating (“nanocoating”) is multifunctional in various sectors, making it possible to control surface properties including permeability, hydrophilicity, and stiffness^1, 2^. It has been employed for anti-fouling and anti-corrosion coating of ships^3^, hard and anti-moisture coating of automotives^4^, and antimicrobial coating of biomedical devices^5^ and orthopedic implants^6^. Glass and plastic commodities are also coated with nanomaterials for anti-wetting^7^, -fogging^8^, or -bacterial property^9^. Nanocoating has been applied to even individual living cells for endowing the cells with exogenous properties at the single-cell level^10^. However, most of these coating strategies require stringent selection of materials and methods, because the mutually specific interactions between the coating materials and target substrates are the decisive factor in the formation of stable coatings. On the other hand, material-independent coating has recently emerged as a powerful tool in the implementation of nanocoating to various substrates by utilizing the unique strong adhesive property. In this approach, the mechanistic essence of bioadhesive phenomena, such as byssus of *Mytilus* species^11^, plant lignin, and leather tanning^12^, is chemically extracted and applied for universal nanocoating. For example, tannic acid (TA) and its derivatives make stable metal-organic coordination complex (MOC) with transition or lanthanide metal ions, especially Fe(III), and the MOC nanofilms form rapidly upon mixing of Fe(III) and TA solutions^13–17^. The material-independent, universal Fe(III)-TA nanocoating has been demonstrated with a multitude of substrates, including planar and particulate ones^13–16^, viruses^18^ and living cells^19–21^, and dentinal tubules^22^. The Fe(III)-TA-MOC also has recently been utilized for biphasic interfacial film formation, such as hollow microcapsules in the water-oil biphasic system^23^.

Although the Fe(III)-TA nanocoating ensures film uniformity for nano/micrometer-sized objects, it is a formidable task to coat bulk or water-floating substrates, because the coating method has been limited to diffusion-driven, supramolecular complex formation in solution. The two coating components (Fe(III) and TA) undergo considerably rapid bond formation in the aqueous solution, and the coating efficiency is, therefore, critically subject to mixing conditions, such as stirring intensity and reaction-vessel size. Moreover, it would demand a huge amount of chemicals to submerge entire substrates in the coating solution, which hinders its wide applications in daily life and industrial sectors. Considering these limitations of immersive nanocoating, we envisioned that spray nanocoating would be a practical and powerful alternative; it has been used to form nanofilms on a variety of substrates, having two-dimensionally textured morphologies or three-dimensionally arbitrary shapes, exemplified by spray coating of layer-by-layer multilayers^24^ and polydopamine^25^. In the spray coating, the coating characteristics, such as morphology, thickness, and chemical composition, are readily modulated by changing coating parameters including material concentration, spraying duration, and resting time. In addition, spray coating is much more time-efficient compared with the immersive nanocoating, because spraying and washing steps generally take less than 10 seconds, and the cross-contamination of suspensions is hardly found^26^. In this work, we demonstrate that stable Fe(III)-TA-MOC nanofilms are formed rapidly by spraying of Fe(III) and TA solutions, and apply the developed coating strategy to bulk commodity goods, including shoe insoles and fruits.

**Figure 1:**
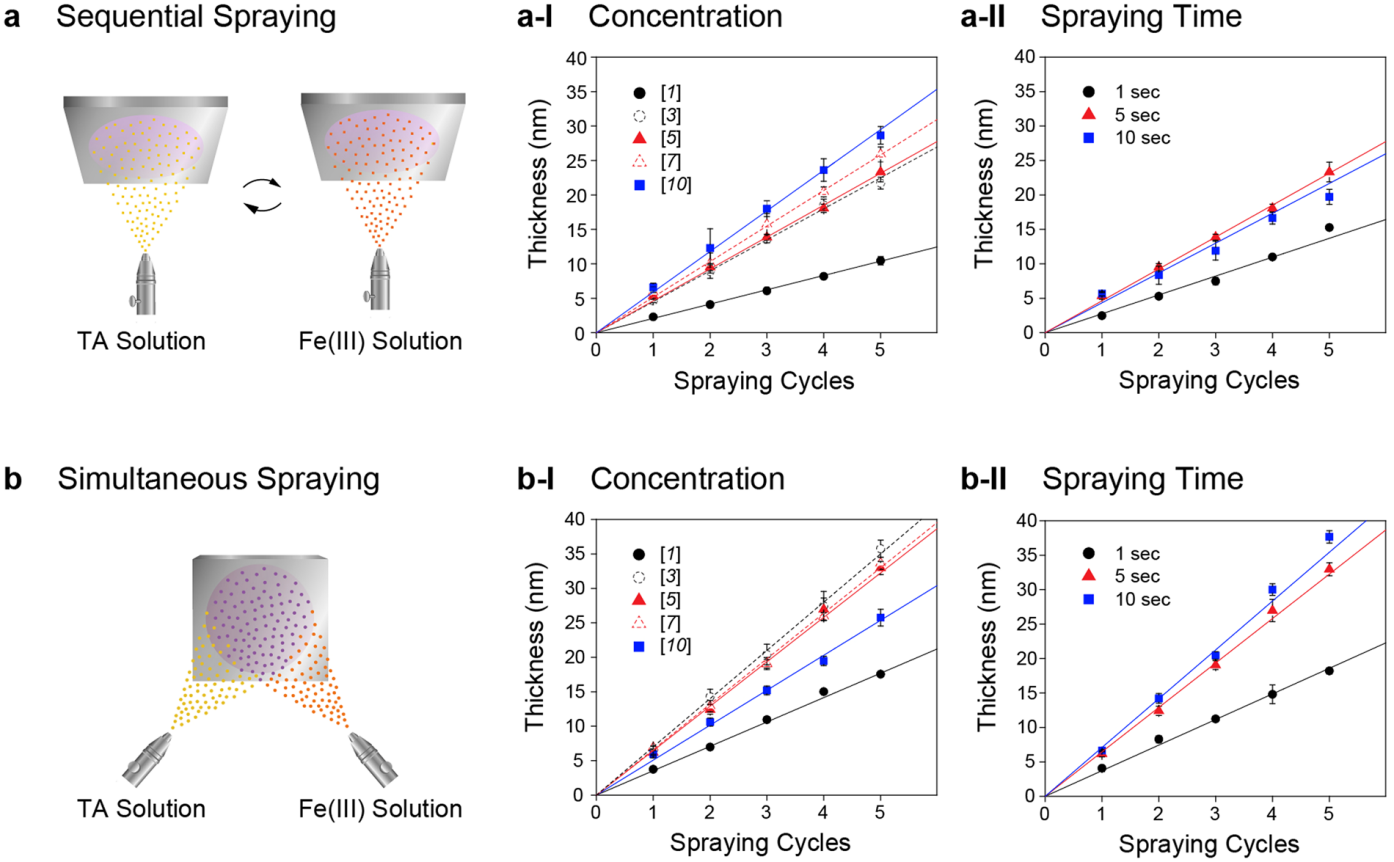
Schematic illustration of Fe(III)-TA spray coating and film thicknesses. (**a**) Sequential (SQ) spraying. (**b**) Simultaneous (SM) spraying. The ellipsometric thicknesses of Fe(III)-TA-MOC films on gold, formed with various concentrations of coating materials (Fe(III) and TA) and different spraying times, are presented as graphs *vs*. the number of spraying cycles. [*n*] in the graphs indicates that the concentration of Fe(III) is *n* mM, and that of TA is also *n* mM.

The coating characteristics were investigated with gold-coated silicon wafers as a substrate. Fe(III) and TA solutions were prepared simply by dissolving FeCl_3_ and TA in deionized (DI) water, respectively. The Fe(III) and TA concentrations were varied to be 1, 3, 5, 7, or 10 mM. In this paper, we, for simplicity, denote [*n*] as a system that uses *n* mM of Fe(III) and *n* mM of TA for spray coating. We used a commercially available dual-action airbrush equipped with 0.3-mm nozzle operated by gravity feeding system, and the pressure was supplied by air at 10.5 L/min (YAMATO COMP, Korea). In the sequential (SQ) spraying, the spraying was started with TA for facile priming, followed by spraying of the Fe(III) solution (Fig. 1a). Each spraying lasted for 5 sec, and the gold substrate was washed intensively with DI water and dried under a stream of argon after the cycle (*i.e*., SQ spraying of TA and Fe(III) solutions). The cycle was repeated up to 5 times.

The ellipsometric measurements showed that the film thickness increased linearly with the number of spraying cycles, reminiscent of layer-by-layer assembly, and the thickness increase per cycle was concentration-dependent (Fig. 1a-I). The slopes in Fig. 1a-I for SQ spraying were calculated to be 2.1 for [*1*], 4.5 for [*3*], 4.6 for [*5*], 5.2 for [*7*], and 5.8 nm/cycle for [*10*]. The film thicknesses after 5 cycles ([*n*]_5_) were 10.5 for [*1*]_5_, 21.7 for [*3*]_5_, 23.3 for [*5*]_5_, 25.9 for [*7*]_5_, 28.6 nm for [*10*]_5_, respectively. The variation in spraying time indicated that 5 sec of SQ spraying formed thick films (Figure 1a-II): 2.8 for 1-sec spraying, 4.6 for 5-sec spraying, and 4.1 nm/cycle for 10-sec spraying in the case of [*5*]. It was also found that the Fe(III)-TA-MOC film was formed by simultaneous (SM) spraying of TA and Fe(III) solutions, which would significantly reduce the coating steps and labor especially in practical, daily-life settings (Fig. 1b). In contrast to the SQ spraying, we did not observe any noticeable increase in thickness above [*3*] ([Fe(III)] = 3 mM; [TA] = 3 mM) in the SM spraying, and the film thickness decreased for [*10*] (Fig. 1b-I). The scanning electron microscopy (SEM) analysis of SM-spraying samples showed that micrometer-sized big agglomerates of Fe(III)-TA-MOC were deposited on the gold substrates for [*10*], and the films became heterogeneous for more than [*5*] (Figure S1a). We thought that, in the SM spraying, the agglomeration had occurred in the air before the aerosols reached the substrate surface due to the rapid formation of coordination bonds between Fe(III) and TA. The consumption of coating materials in the air also would decrease the effective concentrations of Fe(III) and TA for film formation, leading to the decrease in thickness for [*10*]. The atomic force microscopy (AFM) images of the film areas that did not contain the big agglomerates indicated the Fe(III)-TA-MOC films were homogeneous and uniform, mainly composed of nanometer-sized particulates (Figure S1b). Accordingly, the root-mean-square (RMS) roughness ranged in 2–2.5 nm for [*1*] to [*7*] and increased to 3.5 nm for [*10*] in the given area. In terms of spraying time, the 5-sec SM spraying gave thicker films (35.7 for SM(*5*)-[*3*]_5_ and 33.0 nm for SM(*5*)-[*5*]_5_; SM(*t*), *t* = spraying duration) than the 5-sec SQ spraying with [*10*] (28.6 nm for SQ(*5*)-[*10*]_5_) (Figure 1b-II). In the SM spraying, longer spraying time increased the film thickness (*e.g*., 37.7 nm for SM(*10*)-[*5*]_5_), but not greatly for spraying of longer than 5 sec. The SM(*5*)-[*5*]_5_ film was characterized as a representative by Fourier-transform infrared (FT-IR) spectroscopy and X-ray photoelectron spectroscopy (XPS). The FT-IR spectrum showed the intense bands at 1718 (C = O stretching), 1608 (υ C-O from aromatic ring-C-O), and 1442, 1335 and 1220 cm^−1^ (catechol ring vibration), corresponding to the chemical structure of TA (Figure S2a)^27, 28^. The XPS spectra (Figure S2b) confirmed the presence of Fe(III) in the film with peaks at 710 and 724 eV (Fe 2p_1/2_ and 2p_3/2_), and the disappearance of peaks around 91 eV (for gold) indicated that the SM(*5*)-[*5*]_5_ film was stable against X-ray irradiation, and the film thickness was greater than the sampling depth of XPS (8~10 nm)^29^. In addition, the stoichiometry of Fe(III) and TA in the films was calculated to be about 4:1 based on narrow-scan XPS spectra, which was similar to the Fe(III)-TA-MOC films formed by multi-step LbL deposition^14^ and biphasic interfacial assembly^23^.

**Figure 2:**
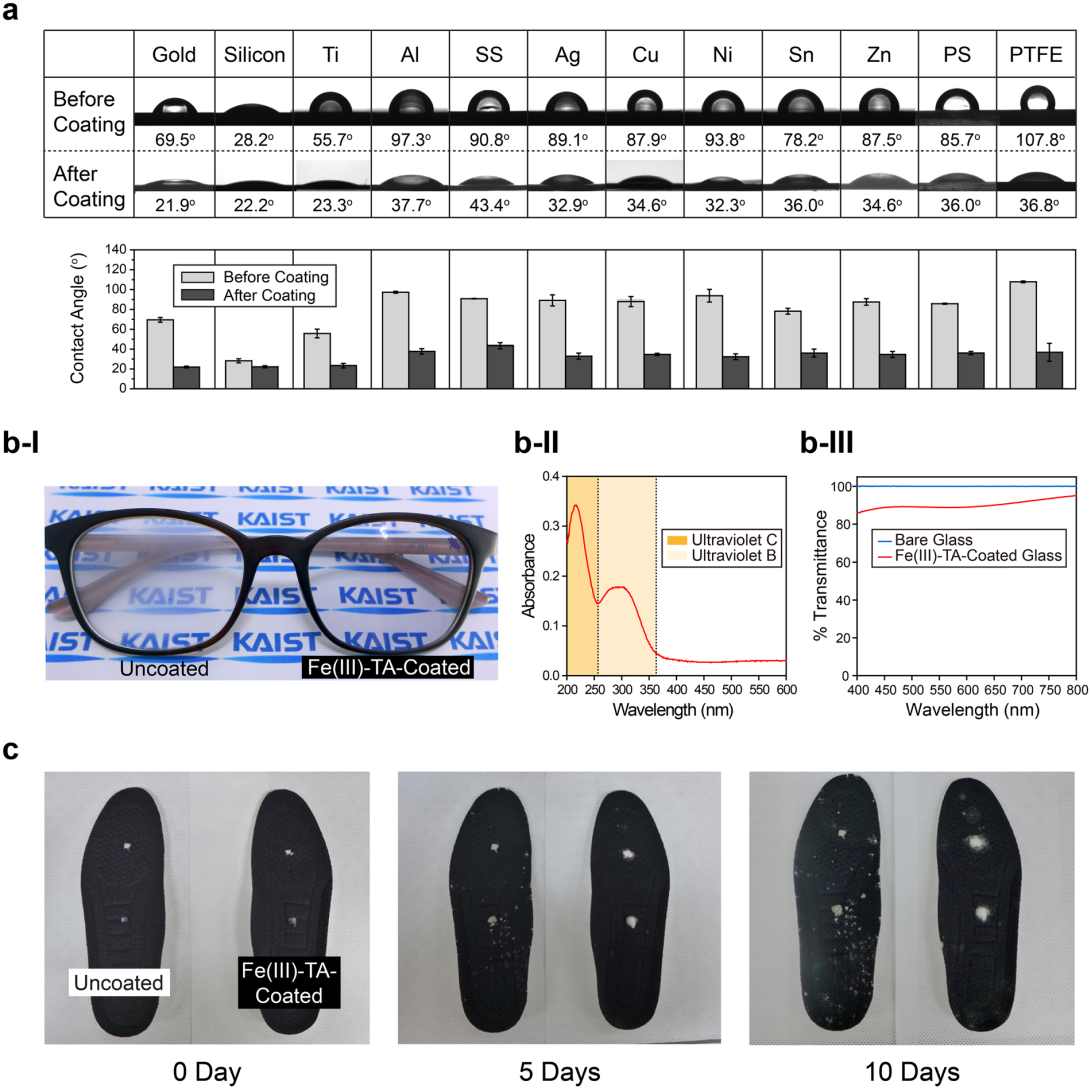
Characteristics and properties of spray-coated Fe(III)-TA-MOC films. (**a**) Material-independent coating characteristics. (top) Optical photographs of water droplets on various substrates before and after SM(*5*)-[*5*]_5_ film formation. Silicon: Si/SiO_2_; SS: stainless steel. (bottom) Graphs of water contact angles before and after SM(*5*)-[*5*]_5_ film formation. (**b**) Anti-fogging, UV-protective, and transparent Fe(III)-TA-MOC films. (b-I) Photograph of glasses with uncoated and Fe(III)-TA-coated lenses. The logos of KAIST are clearly seen though the Fe(III)-TA-coated lens. (b-II) UV absorbance of Fe(III)-TA-coated quartz. (b-III) Percent transmittance of bare and Fe(III)-TA-coated quartz in the visible light region. **c**, Anti-fungal property of Fe(III)-TA-MOC films. Optical photographs of uncoated and Fe(III)-TA-coated shoe insoles after inoculation of *T. rubrum* (white). Also see Figure S4 for clearer comparison with enlarged images.

The material independency of Fe(III)-TA-MOC spray nanocoating was confirmed with various substrates, including gold (Au), silicon (Si/SiO_2_), titanium oxide (TiO_x_; Ti), aluminum (Al), silver (Ag), copper (Cu), nickel (Ni), tin (Sn) and zinc (Zn) foils, stainless steel (SS), polystyrene (PS), and polytetrafluoroethylene (PTFE). The water static contact angles were measured before and after SM(*5*)-[*5*]_5_ coating for each substrate (Fig. 2a). All the substrates tested became hydrophilic (contact angle ~ 35°) regardless of their hydrophobicity before spray coating. Even PTFE with extremely low surface energy of 18.5 mN/m could be coated with Fe(III)-TA-MOC films. In addition to the material independency, the Fe(III)-TA-MOC film was found to be both anti-fogging and UV-protective (Fig. 2b). One lens of plastic glasses, made of poly(methyl methacrylate) (PMMA), was spray-coated, and the other kept intact. In the high humidity environment, only coated lens displayed the anti-fogging property clearly, presumably from the increased surface-hydrophilicity (Fig. 2b-I). The UV-Vis spectrum of the Fe(III)-TA-MOC film on quartz showed distinct absorption bands at UV-B and UV-C regions (Figure 2b-II and Figure S3), and the transmission was up to 90% at visible light region (Figure 2b-III). The Fe(III)-TA-MOC film was also anti-fungal. We chose *Trichophyton rubrum*, which is a prevailing fungus causing the athlete’s foot (tinea pedis), as a model^30^. The commercial shoe insoles were coated with SQ(*5*)-[*10*]_5_ films and immersed for 10 min in the Sabouraud broth solution to supply nutrients for colonization, followed by inoculation of *T. rubrum* at the top and the middle positions of the insole and incubation at 25 °C (Fig. 2c). The result was noteworthy that the Fe(III)-TA-MOC coating strongly prohibited the growth and spreading of *T. rubrum* up to 10 days (Figure S4 for the enlarged photo taken after 10 days of incubation). Taken together, these results clearly confirmed that the spray coating of Fe(III) and TA was rapid (<5 sec), thickness-controllable, and material-independent. Of more importance is that the spray coating developed can be applied to bulk substrates, such as glasses and shoe insoles; the film properties―anti-fogging, UV-filtering, transparent, and anti-microbial―would promise practical potential in the spray-based system development for commodity products.

**Figure 3:**
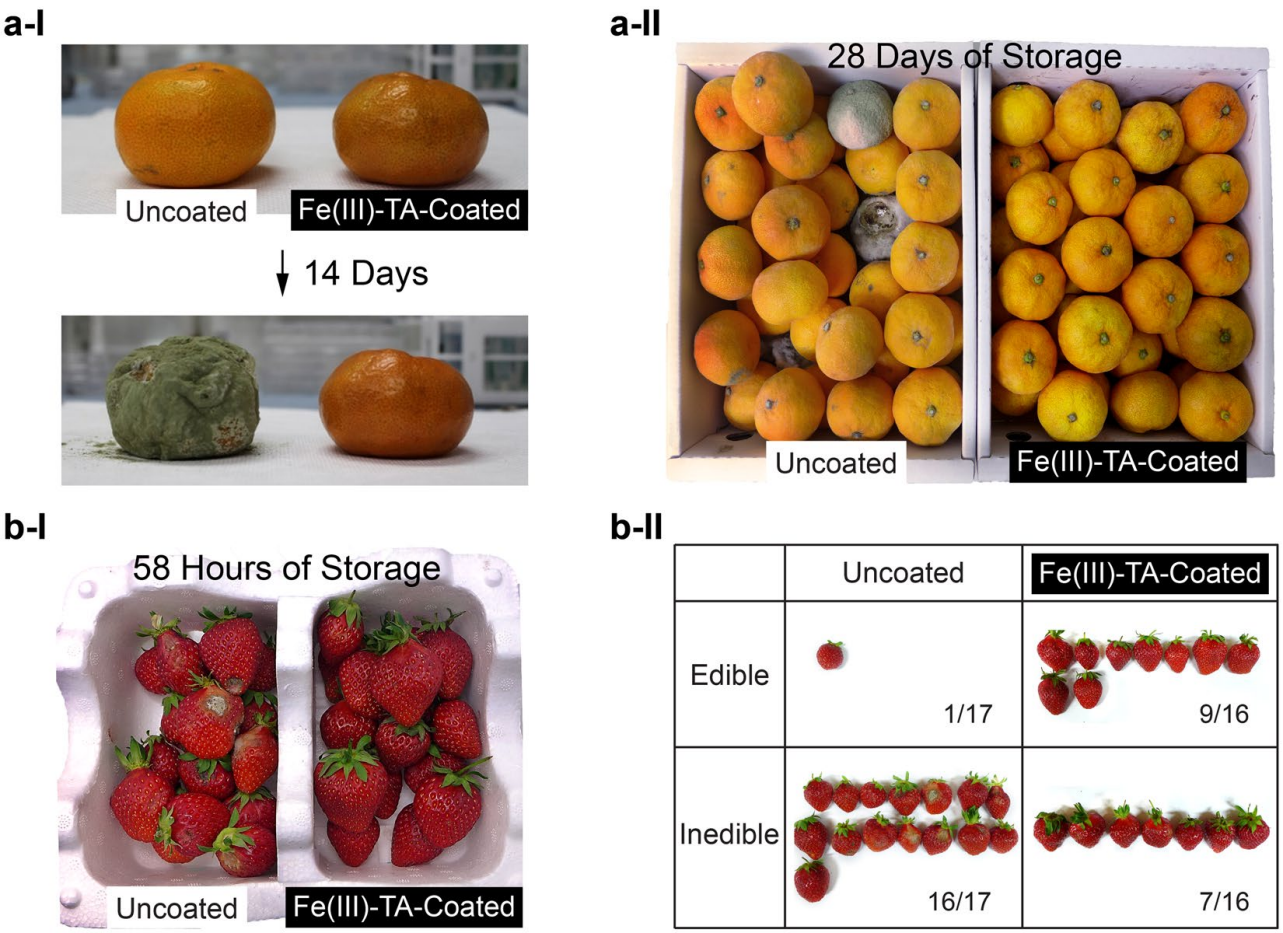
Edible Fe(III)-TA spray coating that prolongs post-harvest shelf-life. (**a**) Mandarin oranges. (a-I and II) Optical photographs of uncoated and Fe(III)-TA-coated mandarin oranges after storage at room temperature. (**b**) Strawberries. (b-I) Optical photograph of uncoated and Fe(III)-TA-coated strawberries after storage. (b-II) Statistical analysis of edible strawberries from the sample in (b-I). Also see Figure S5 and S6 for clearer comparison with enlarged images.

Edible coating of fruits and vegetables has drawn much attention in the food and agricultural industry, because it could not only prolong postharvest shelf-life of produce against external changes of environment but also provide additional nutrients to be useful in human health^31, 32^. For example, Omenetto *et al*. have recently demonstrated the edible coating of strawberries and bananas by dip coating with silk fibroin, but it involves multi-cycled dip coatings and long annealing (up to 12 h)^33^. TA is a plant-derived polyphenol, which has been used as food, cosmetic, and pharmaceutical additives^34^, and, therefore, is eco-friendly. Fe(III) is also one of the components in nutritional supplements. We, therefore, envisioned that the Fe(III)-TA-MOC spray coating would have practical promise for edible coating of fruits and vegetables because of distinctive advantages: from the coating-method standpoint, the spray coating, in addition to its rapidity, allows us to avoid time-consuming repetitive processes and to manage the wide variety of parameters for optimized edible coating; from the coating-material standpoint, iron(III) chloride (ID Code: 7705-08-0) and TA (ID Code: 1401-55-4)^35^ are recognized to be safe under FDA regulation, although detailed studies on toxicity of coated fruits might be needed^36^. As a demonstration, mandarin oranges (grown in Jeju Island, Korea) were chosen to investigate the effect of Fe(III)-TA-MOC spray nanocoating on their postharvest shelf-life (Fig. 3a). The uncoated and coated (SQ(*5*)-[*10*]_5_) mandarin oranges were placed on a Kimwipe under ambient conditions (25 °C). A representative result was shown in Fig. 3a-I: the coated mandarin orange maintained its intact surface and shape for 14 days, but the uncoated one was completely rotten with greenish mold. Statistical studies were also performed with dozens of mandarin oranges in a box, and, for practical applications, washing for decolorization was done with tap water, and drying with a spinning fan. The mandarin oranges without any wax coating were purchased directly from a farmer. Commercially available spray systems with 0.2-mm nozzle (Ultimate Spray Station HV3900, USA) were used for SM(*3*) spraying of [*5*]. After 28 days of storage at 25 °C in the daily-life setting, 27% of the uncoated mandarin oranges (10 out of 37) were rotten and covered with molds, while none of the coated mandarin oranges was rotten (Figure 3a-II).

A field test was also performed at a farm for strawberry coating, because they become rapidly softer after harvest. Strawberries on trees (before harvest) were spray-coated via SM-[*5*] (spraying duration per strawberry <3 sec) and subsequently washed with a spray of tap water, at around 5–6 pm in the afternoon. The sprayed strawberries, along with the un-sprayed ones as a control, were harvested at 9 am on the next day (around the last picking time for strawberries at the strawberry farms in Korea). The spraying did not change the taste and texture of strawberries at the blind test. A set of strawberries were stored in a Styrofoam box at ambient temperature (25 °C) and humidity (32–45% relative humidity). After 58 h of storage, we observed that un-sprayed strawberries became rotten with white mold, but the sprayed strawberries kept intact (Fig. 3b-I and Figure S5 for the enlarged photo). The strawberries were divided into edible and inedible ones: while 56.3% of the sprayed strawberries were edible, only 6.3% of the un-sprayed looked edible (Figure 3b-II and Figure S6 for the enlarged photo). We also investigated the effects of spraying on growth of strawberries. After SM spraying in a strawberry field, about 30 strawberries with different sizes and colors were labeled on the stems with a tape, and their growth was monitored for 3 days with uncoated ones as a control. The sprayed strawberries properly grew to be good and ripe, compared with the un-sprayed ones, indicating that the spraying did not alter nor suppress the growth. In addition, we observed that the microbial effects of the Fe(III)-TA-MOC spraying lasted at least for two days: the strawberries, harvested 2 days after spraying, still showed enhanced resistance against molds (data not shown).

In summary, we developed a rapid spray coating of Fe(III)-TA-MOC for bulk objects, such as glasses, shoe insoles, and fruits. The 5 second of spray coating forms a uniform film with about 5 nm in thickness, and the film thickness can be varied in a controlled, linear fashion by the number of coating cycles. Especially, the simultaneous spraying of Fe(III) and TA―desired for daily-life bulk coating―leads to the uniform films, the thickness of which is varied by the concentration of coating materials and the spraying time. As a demonstration, we coated mandarin oranges and strawberries to increase their shelf-life after harvest while maintaining freshness. The on-site studies in the strawberry field clearly showed the direct applicability of the spraying protocol developed herein to edible coating of fruits and vegetables. We also believe that this rapid and simple method for forming stable films on bulk substrates would find its applications in various industrial sectors other than agricultural technology.

## Methods

### Spray coating of Fe(III)-TA-MOC on gold

Prior to use, gold substrates (2 cm × 2 cm) were immersed in ethanol, sonicated for 15 min, rinsed with deionized (DI) water, and dried under a stream of argon gas. The solutions of TA (1, 3, 5, 7, or 10 mM) and Fe(III) (1, 3, 5, 7, or 10 mM) were freshly prepared with DI water, and a pair of the solutions ([*1*], [*3*], [*5*], [*7*], or [*10*]) were placed in each, respectively. The angle between two air brushes and the distance between the air brush and the substrate were fixed to 35° and 20 cm, respectively. For sequential (SQ) spraying, the spraying was started with TA, followed by spraying of the Fe(III) solution. Each spraying lasted for 1, 5, or 10 sec. For simultaneous (SM) spraying, the TA and Fe(III) solution were sprayed at the same time for 1, 5, or 10 sec. The coated substrate was washed intensively with DI water and dried under a stream of argon, followed by the ellipsometric thickness measurements, after the cycle. The cycle was repeated for a desired number of iteration.

### Shoe insoles

Sabouraud broth was made by adding 10 g of peptone and 40 g of glucose to 1 L of DI water, and sterilized by autoclaving at 120 °C for 20 min. Commercial shoe insoles were coated with the SQ(*5*)-[*10*]_5_ films (spraying time: 5 sec, coating solution: [*10*], number of cycles: 5) and then immersed in the broth solution for 10 min to supply nutrients for colonization. Agar pieces of *Trichophyton rubrum* (KCTC 6375) were taken from an agar plate, which had *T. rubrum* colonies, and placed on the top and middle positions of the insoles. The shoe insoles were placed in a slightly opened plastic box to prevent desiccation of the broth, and incubated at 25 °C.

### Mandarin oranges

The solutions of TA (10 mM) and Fe(III) (10 mM) were freshly prepared with DI water, and poured into each container of the spray brushes. Mandarin oranges, which were purchased directly from a farmer and not coated with any waxes, were coated with SQ(*5*)-[*10*]_5_ films (spraying time: 5 sec, coating solution: [*10*], number of cycles: 5). After each spraying cycle, they were cleaned with DI water and dried under a stream of argon. For bulk-scale coating, the SM spraying was employed with 2.0-mm nozzle spray devices (Ultimate SparyStation HV3900 KR, Earlex). As-purchased mandarin oranges were placed on a round-shaped stainless strainer for ease of washing process, and coated with SM(*3*)-[*5*] films (spraying time: 3 sec, coating solution: [5]). The coated mandarin oranges were washed with tap water, dried under a spinning fan, and placed in a cardboard box for characterizations.

### Strawberries

Ultimate SparyStation HV3900 KR (Earlex) was used for SM spraying onto strawberries (Figure S4). The solutions of TA (5 mM) and Fe(III) (5 mM) were freshly prepared with tap water in each container (1 L) of the spray device, respectively. A backpack-sized agricultural sprayer (5 L) was also filled with tap water for washing. The TA and Fe(III) solutions were sprayed simultaneously onto the strawberries on trees for 3 sec per strawberry, and the distance between the spray nozzle and strawberries was about 20 cm. The strawberry trees were subsequently washed with tap water by using the agricultural sprayer. The coating was performed at around 5–6 pm in the afternoon, and the coated strawberries, along with the uncoated ones as a control, were harvested at 9 am on the next day. Two layers of strawberries were stored in a Styrofoam box at ambient temperature (25 °C) and humidity (32–45% relative humidity).

### Data availability

All relevant data are available from the authors.

## Electronic supplementary material

Supplementary Information
