# Publisher Correction: Antimicrobial spray nanocoating of supramolecular Fe(III)-tannic acid metal-organic coordination complex: applications to shoe insoles and fruits

**DOI:** 10.1038/s41598-018-37133-1

**Published:** 2018-12-14

**Authors:** Ji Hun Park, Sohee Choi, Hee Chul Moon, Hyelin Seo, Ji Yup Kim, Seok-Pyo Hong, Bong Soo Lee, Eunhye Kang, Jinho Lee, Dong Hun Ryu, Insung S. Choi

**Affiliations:** 1 Center for Cell-Encapsulation Research, Department of Chemistry, KAIST, Daejeon, 34141 Korea; 2 Startup KAIST, KAIST, Daejeon, 34141 Korea; 3 HC Lab, 235 Creation Hall, 193 Munji Road, Daejeon, 34051 Korea

Correction to: *Scientific Reports* 10.1038/s41598-017-07257-x, published online 1 August 2017

The original HTML version of this Article contained errors in the spelling of the authors Ji Hun Park, Hee Chul Moon, Ji Yup Kim, Bong Soo Lee & Dong Hun Ryu, which were incorrectly given as Ji Park, Hee Moon, Ji Kim, Bong Lee & Dong Ryu respectively.

These errors have now been corrected in the HTML version of this Article; the PDF version was correct from the time of publication.

