# Supplementary material for: Antimicrobial spray nanocoating of supramolecular Fe(III)-tannic acid metal-organic coordination complex: applications to shoe insoles and fruits
Source: Sci Rep. 2017 Aug 1;7:6980. doi: 10.1038/s41598-017-07257-x (PMC5539098; doi:10.1038/s41598-017-07257-x)
Supplement: Supplementary file 1 — Supplementary Information [file 41598_2017_7257_MOESM1_ESM.pdf]

SUPPLEMENTARY INFORMATION

Antimicrobial spray nanocoating of supramolecular Fe(III)-tannic acid metal-organic coordination complex: applications to shoe insoles and fruits

Ji Hun Park[1], Sohee Choi[1†], Hee Chul Moon[1†], Hyelin Seo[1], Ji Yup Kim[1], Seok-Pyo Hong[1,3], Bong Soo Lee[1,3], Eunhye Kang[1], Jinho Lee[2], Dong Hun Ryu[3], and Insung S. Choi[1]*

[1]Center for Cell-Encapsulation Research, Department of Chemistry, KAIST, Daejeon 34141, Korea. [2]Startup KAIST, KAIST, Daejeon 34141, Korea. [3]HC Lab, 235 Creation Hall, 193 Munji Road, Daejeon 34051, Korea.

[†]These authors contributed equally to this work.

*e-mail: ischoi@kaist.ac.kr

CONTENTS

- **Experimental section**.
- **Figure S1.** SEM and AFM images of Fe(III)-TA nanofilms on gold, formed by SM spraying.
- **Figure S2.** FT-IR and XPS spectra of SM(5)-[5]$_5$ films on gold.
- **Figure S3.** Photograph of uncoated and Fe(III)-TA-coated quartz cells.
- **Figure S4.** Enlarged photograph of uncoated and Fe(III)-TA-coated shoe insoles.
- **Figure S5.** Enlarged photograph of uncoated and Fe(III)-TA-coated strawberries.
- **Figure S6.** Enlarged photograph of edible and inedible strawberries.

Experimental section

Materials. Tannic acid (TA, Sigma) and iron(III) chloride hexahydrate ($FeCl_3 \cdot 6H_2O$, 99%, Aldrich) were used as received. Silicon wafers were purchased from Wafer Korea, Inc.. Gold and titanium dioxide (TiO_2) substrates were prepared by coating each component onto a silicon wafer with the thickness of 100 nm by using a thermal evaporator. Stainless steel (Fe:Cu:Ni; 70:19:11 wt%), Ag foil (99.998%), Cu foil (99.95%), Ni foil (99.5%), Sn foil (99.8%), Zn foil (99.98%) were purchased from Alfa Aesar. Ultrapure water (18.3 MΩ·cm) from the Human Ultrapure System (Human Corp.) was used. Spray brushes and air compressors were purchased from Yamato Comp. The nozzle size was 300 μm in diameter, and the spray rate was about 10 L/min.

Spray coating of Fe(III)-TA-MOC on gold. Prior to use, gold substrates (2 cm × 2 cm) were immersed in ethanol, sonicated for 15 min, rinsed with deionized (DI) water, and dried under a stream of argon gas. The solutions of TA (1, 3, 5, 7, or 10 mM) and Fe(III) (1, 3, 5, 7, or 10 mM) were freshly prepared with DI water, and a pair of the solutions ([1], [3], [5], [7], or [10]) were placed in each, respectively. The angle between two air brushes and the distance between the air brush and the substrate were fixed to 35° and 20 cm, respectively. For sequential (SQ) spraying, the spraying was started with TA, followed by spraying of the Fe(III) solution. Each spraying lasted for 1, 5, or 10 sec. For simultaneous (SM) spraying, the TA and Fe(III) solution were sprayed at the same time for 1, 5, or 10 sec. The coated substrate was washed intensively with DI water and dried under a stream of argon, followed by the ellipsometric thickness measurements, after the cycle. The cycle was repeated for a desired number of iteration.

Shoe insoles. Sabouraud broth was made by adding 10 g of peptone and 40 g of glucose to 1 L of DI water, and sterilized by autoclaving at 120 ^{o}C for 20 min. Commercial shoe insoles were coated with the SQ(*5*)-[*10*]$_5$ films (spraying time: 5 sec, coating solution: [10], number of cycles: 5) and then immersed in the broth solution for 10 min to supply nutrients for colonization. Agar pieces of *Trichophyton rubrum* (KCTC 6375) were taken from an agar plate, which had *T. rubrum* colonies, and placed on the top and middle positions of the insoles. The shoe insoles were placed in a slightly opened plastic box to prevent desiccation of the broth, and incubated at 25 ^{o}C.

Mandarin oranges. The solutions of TA (10 mM) and Fe(III) (10 mM) were freshly prepared with DI water, and poured into each container of the spray brushes. Mandarin oranges, which were purchased directly from a farmer and not coated with any waxes, were coated with SQ(*5*)-[*10*]$_5$ films (spraying time: 5 sec, coating solution: [10], number of cycles: 5). After each spraying cycle, they were cleaned with DI water and dried under a stream of argon. For bulk-scale coating, the SM spraying was employed with 2.0-mm nozzle spray devices (Ultimate SparyStation HV3900 KR, Earlex). As-purchased mandarin oranges were placed on a round-shaped stainless strainer for ease of washing process, and coated with SM(*3*)-[*5*] films (spraying time: 3 sec, coating solution: [5]). The coated mandarin oranges were washed with tap water, dried under a spinning fan, and placed in a cardboard box for characterizations.

Strawberries. Ultimate SparyStation HV3900 KR (Earlex) was used for SM spraying onto strawberries (Figure S4). The solutions of TA (5 mM) and Fe(III) (5 mM) were freshly prepared with tap water in each container (1 L) of the spray device, respectively. A backpack-sized

agricultural sprayer (5 L) was also filled with tap water for washing. The TA and Fe(III) solutions were sprayed simultaneously onto the strawberries on trees for 3 sec per strawberry, and the distance between the spray nozzle and strawberries was about 20 cm. The strawberry trees were subsequently washed with tap water by using the agricultural sprayer. The coating was performed at around 5-6 pm in the afternoon, and the coated strawberries, along with the uncoated ones as a control, were harvested at 9 am on the next day. Two layers of strawberries were stored in a Styrofoam box at ambient temperature (25 ^{o}C) and humidity (32-45% relative humidity).

Characterizations. Ellipsometric measurements were performed with a Gaertner L116s ellipsometer (Gaertner Scientific Corporation) equipped with a He-Ne laser (632.8 nm) at 70° angle of incidence. A refractive index of 1.46 was used for all substrates. At least five independent samples were analyzed for each spraying condition (concentration or spraying time). Five different points of each sample were measured, and average values (with at least 25 measurements) were recorded. Contact angle measurements were performed using a Phoenix 300 goniometer (Surface Electro Optics Co.) equipped with a video camera. The static contact angles of 2 μL water droplet were measured at four different locations on each sample. Two samples were prepared independently, and the average value was recorded. UV absorbance spectra of Fe(III)-TA-MOC films were acquired from 200 to 800 nm with a quartz cuvette (Hellma Analytics) coated with SQ(5)-[*10*]$_5$ films by using a UV-Vis spectrometer (UV-2550, Shimadzu). FT-IR spectra were obtained with an ALPHA FTIR spectrometer ATR module (Bruker Optics). XPS spectra of Fe(III)-TA-MOC films on gold were acquired from a multi-purpose X-ray photoelectron spectroscope (Sigma Probe, Thermo VG Scientific) with Al-Kα (1486.6 eV) X-ray source and pass energy of 50.0 eV (wide scan) and 20 eV (individual narrow scan). SEM images were acquired with a Sirion FEI XL FEG/SFEG microscope (FEI Co.) with an accelerating voltage of 10 kV after sputter-coating with platinum. AFM experiments were conducted on an Agilent 5500 AFM/SPM Microscope (Agilent Technologies). AFM imaging was performed in air, and typical scans were performed in contact mode with NanoWorld silicon nitride AFM tip (PNP-TR-50, 0.08 N/m of force constant and 17 kHz of resonance frequency).

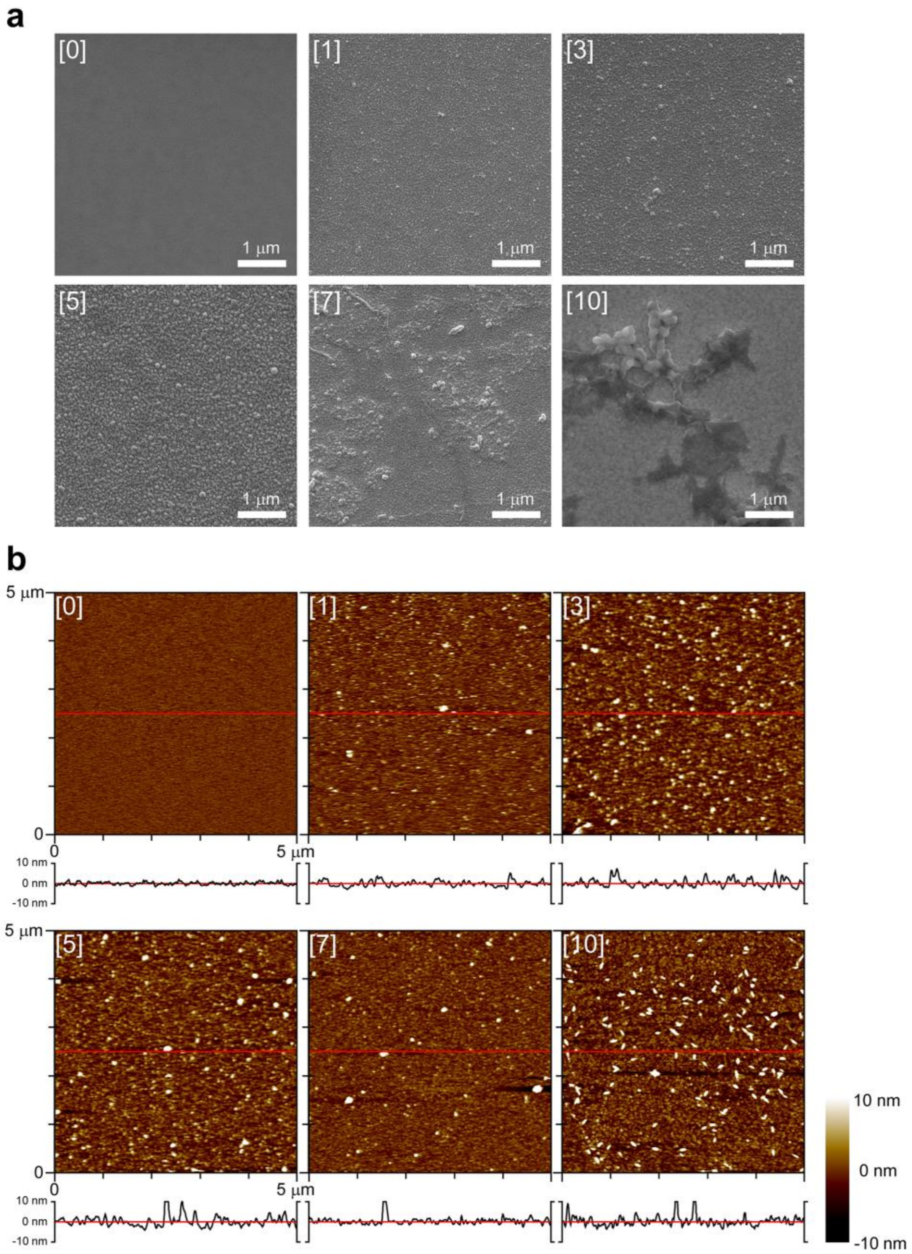

Figure S1 | SEM and AFM characterizations. a, SEM images of Fe(III)-TA-MOC nanofilms formed by SM spraying. **b,** AFM images of Fe(III)-TA-MOC nanofilms formed by SM spraying. [0]: native, uncoated gold substrate.

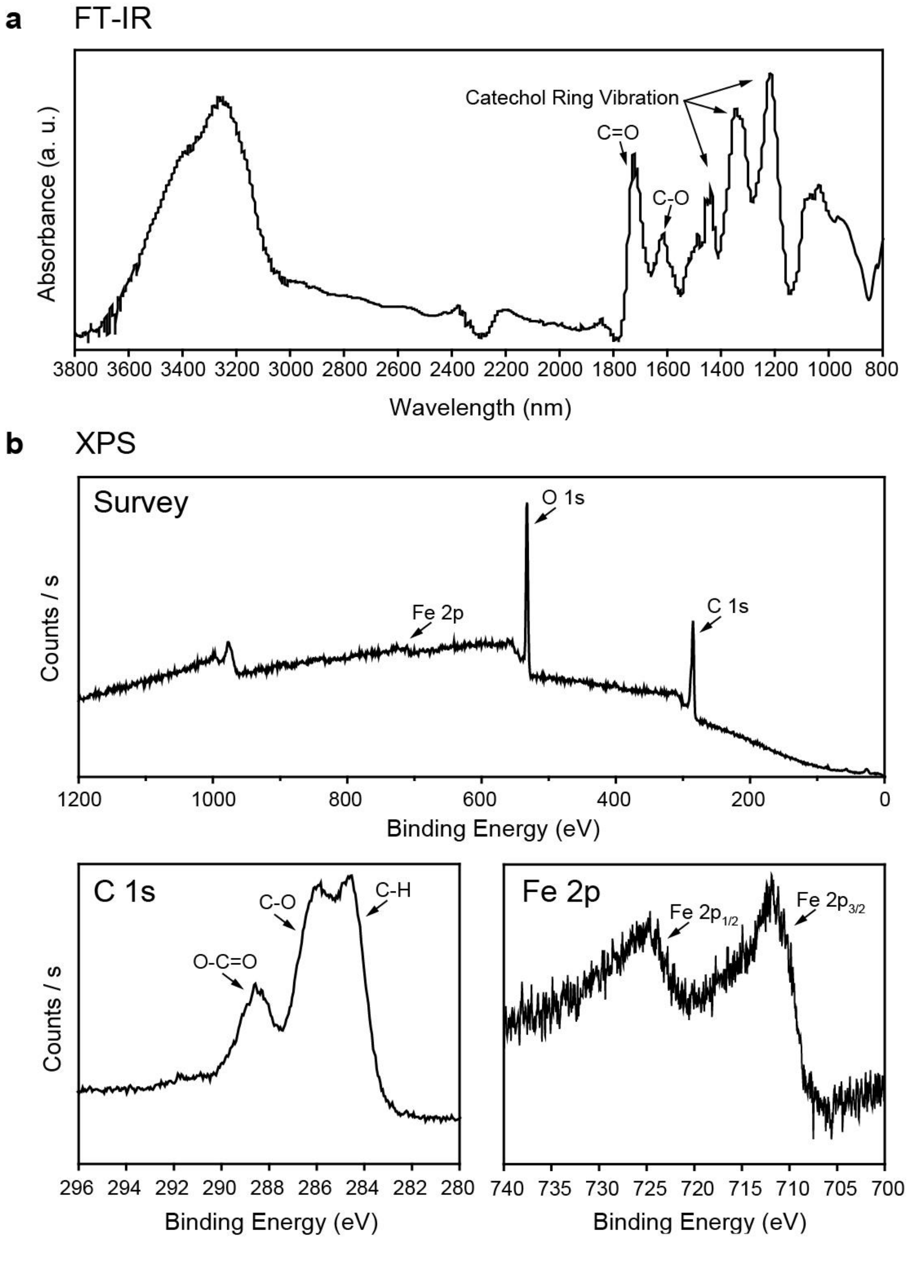

Figure S2 | FT-IR and XPS characterizations. a, FT-IR spectrum of SM(5)-[5]$_5$ films on gold. **b,** XPS spectra of SM(5)-[5]$_5$ films on gold.

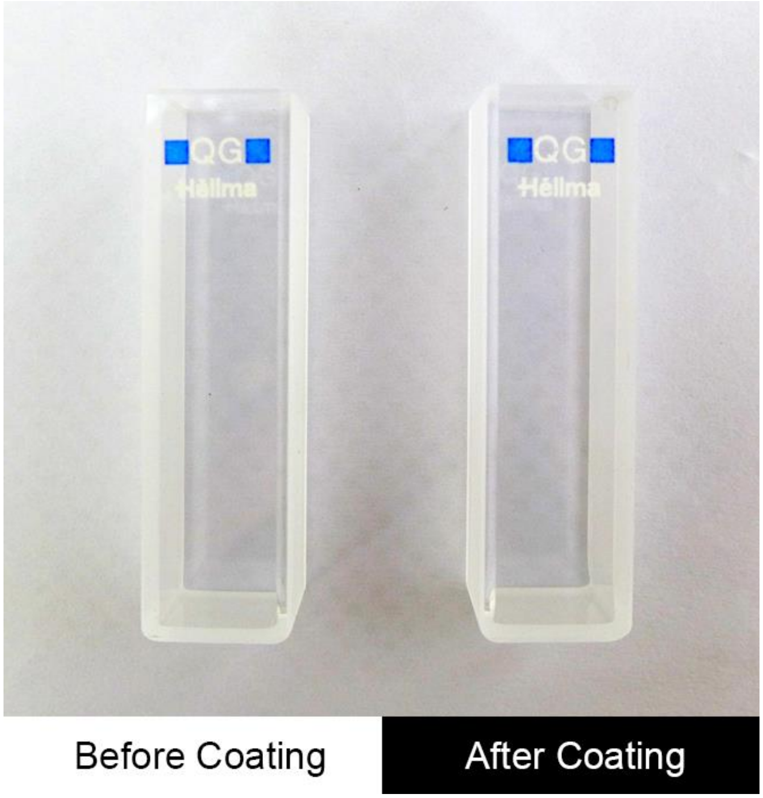

Figure S3 | Coating of quartz cells. Photograph of uncoated and Fe(III)-TA-coated quartz cells.

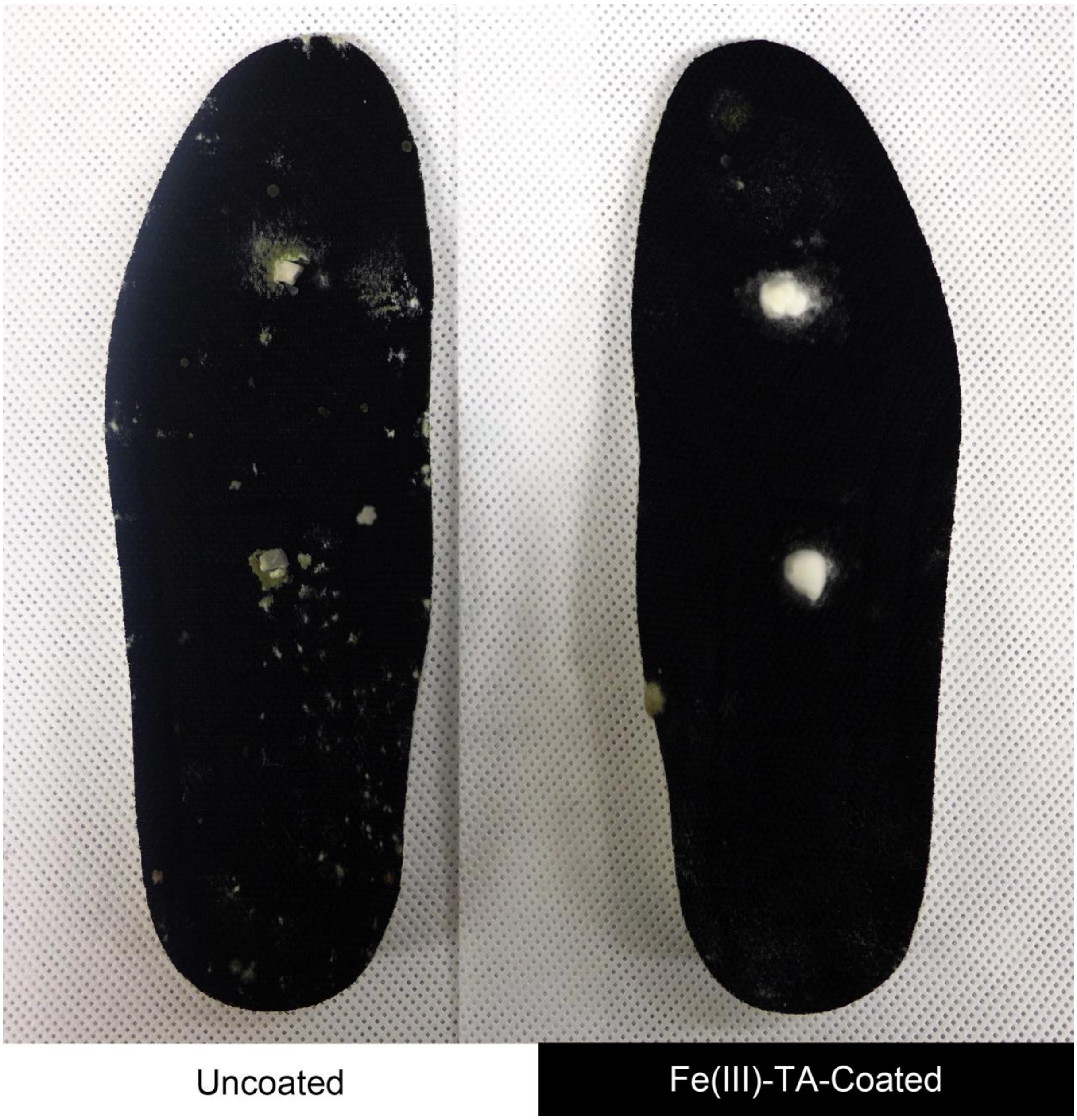

Figure S4 | Anti-fungal Fe(III)-TA-MOC spray coating on shoe insoles. Photograph of uncoated and Fe(III)-TA-coated shoe insoles after the incubation at 25 ^{o}C for 10 days.

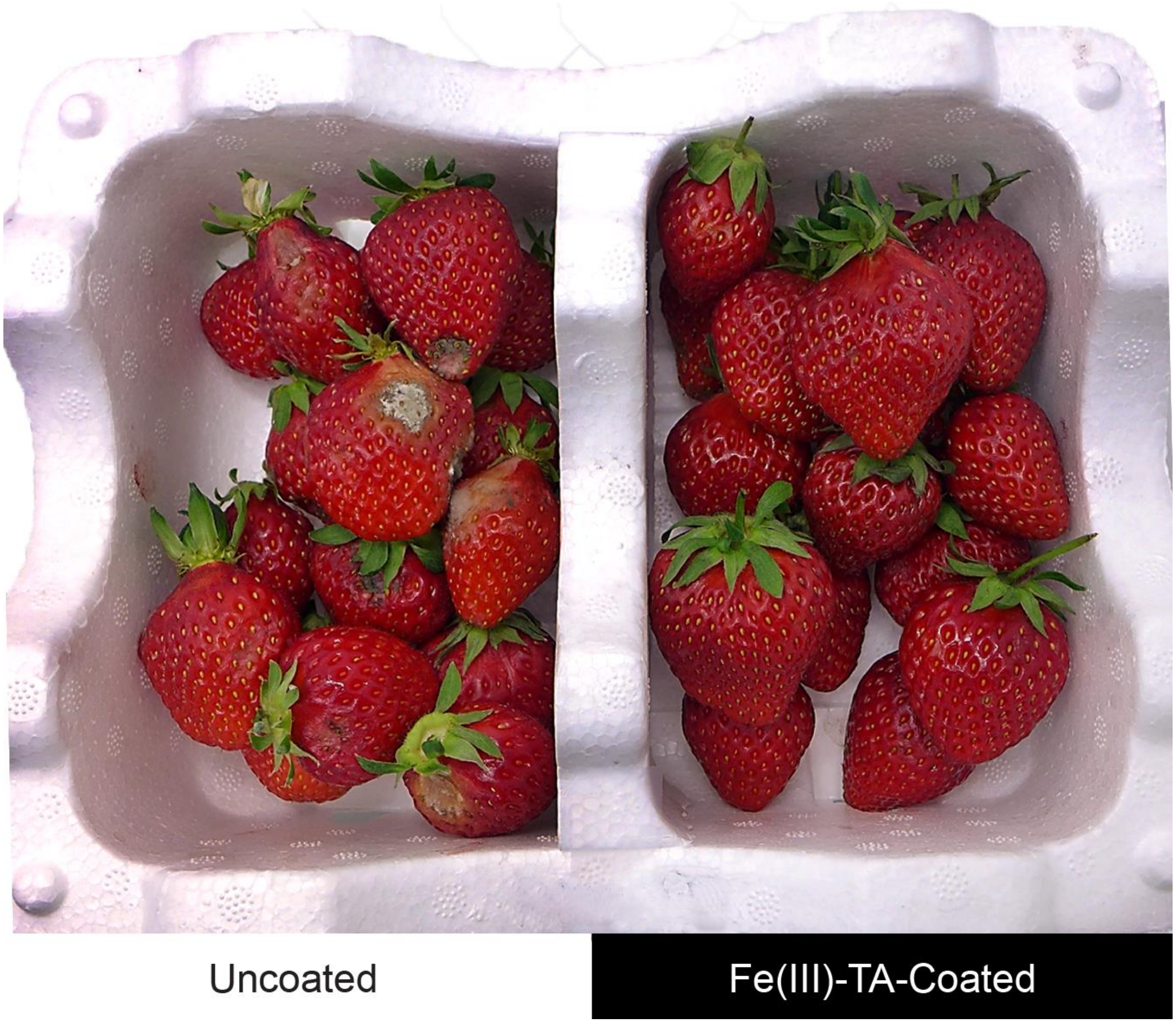

Figure S5 | Edible Fe(III)-TA-MOC spray coating on strawberries. Photograph of uncoated and Fe(III)-TA-coated strawberries after 58 h of storage.

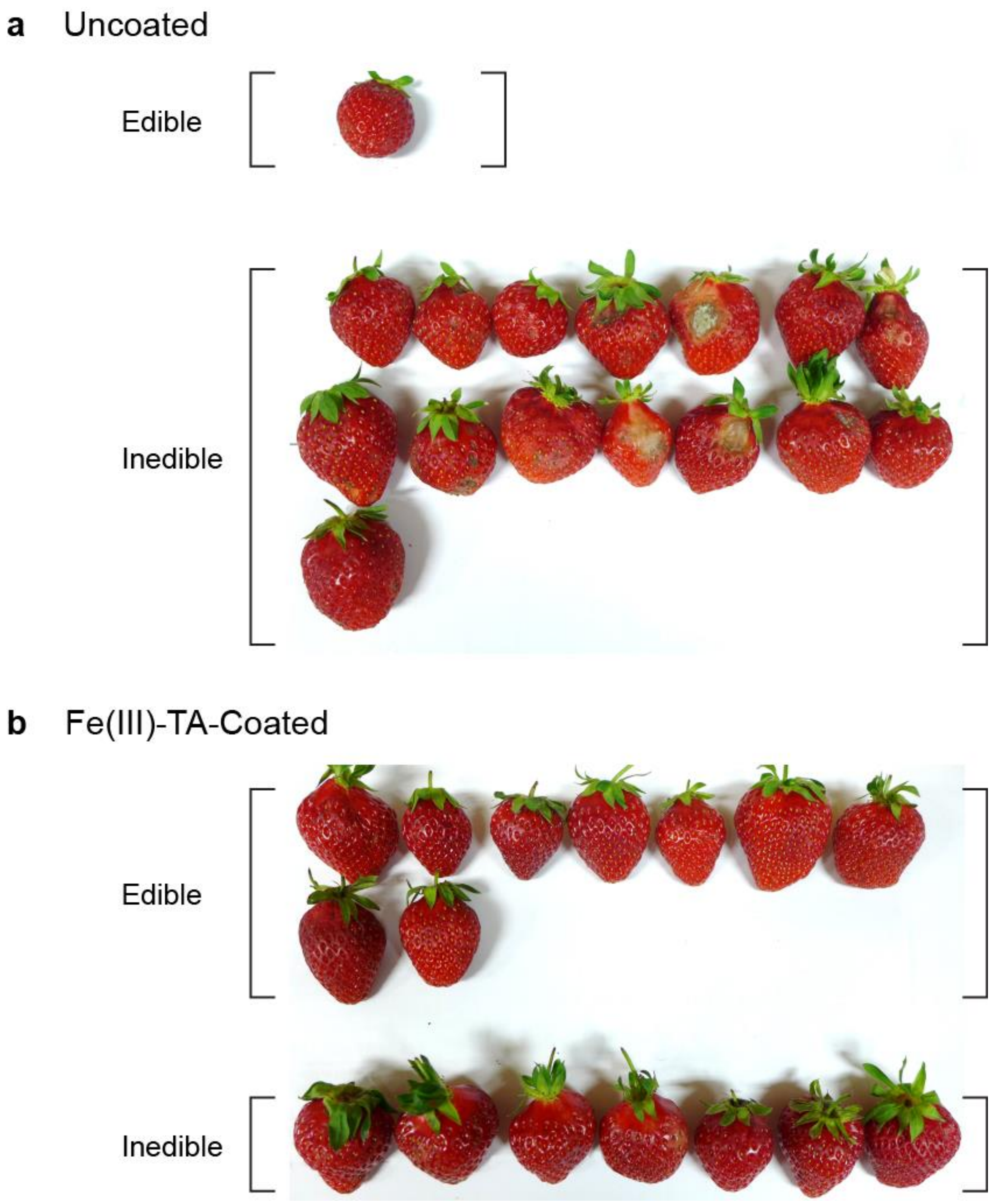

Figure S6 | Edible Fe(III)-TA-MOC spray coating on strawberries. Photographs of (a) uncoated and (b) Fe(III)-TA-coated strawberries after 58 h of storage. The strawberries are divided into edible and inedible strawberries. The inedible strawberries include colorized strawberries with little dents as well as rotten ones. The edible strawberries are selected first (from stringent personal point of view (H.C.M.)), and the others are considered to be inedible.
